# Multicentric prognostic observational study on biomarker profile in immunotherapy-naïve patients with highly active generalized myasthenia gravis (PROGNO-MG): a study protocol

**DOI:** 10.1186/s42466-026-00482-8

**Published:** 2026-05-12

**Authors:** Felix Kohle, Franz Felix Konen, Ulrike Grittner, Daniel Schulze, Stefanie Glaubitz, Christopher Nelke, Christina B. Schroeter, Niklas Huntemann, Peter Trillenberg, Julian Großkreutz, Benedikt Schoser, Charlotte Schubert, Jana Zschüntzsch, Michael Schroeter, Tobias Hegelmaier, Thomas Skripuletz, Jörn Peter Sieb, Tobias Ruck, Sven Meuth, Heinz Wiendl, Christian Wolfgang Keller, Jan D. Lünemann, Sophie Lehnerer, Andreas Meisel, Frauke Stascheit

**Affiliations:** 1 Department of Neurology, Faculty of Medicine, University of Cologne and University Hospital Cologne, Cologne, Germany; 2 Department of Neurology, Hannover Medical School, Hannover, Germany; 3 Institute of Biometry and Clinical Epidemiology, Charité - Universitätsmedizin Berlin, corporate member of, Freie Universität Berlin and Humboldt Universität zu Berlin, Berlin, Germany; 4 Department of Neurology, University Medical Center Göttingen, Göttingen, Germany; 5 Department of Neurology, Medical Faculty, Heinrich Heine University Düsseldorf, Düsseldorf, Germany; 6 Department of Neurology, University Medical Center Schleswig-Holstein, Lübeck, Germany; 7 Department of Neurology, Friedrich-Baur-Institute, Ludwig-Maximilians-University Munich, Munich, Germany; 8 Institute of Neuroimmunology and Multiple Sclerosis (INIMS), University Medical Center Hamburg-Eppendorf, Hamburg, Germany; 9 Department of Neurology, Helios Hanseklinikum Stralsund, Stralsund, Germany; 10 Department of Neurology with, Heimer Institute for Muscle Research, University Hospital Bergmannsheil, Bochum, Germany; 11 Department of Neurology and Neurophysiology, University Hospital Freiburg, Freiburg, Germany; 12 Department of Neurology with Institute of Translational Neurology, University Hospital Münster, Münster, Germany; 13 Department of Neurology with Experimental Neurology, Neuroscience Clinical Resarch Center, Charite Universitatsmedizin Berlin, Berlin, Germany; 14 Berlin Institute of Health at Charité – Universitätsmedizin Berlin, Digital Health Center, Berlin, Germany; 15 Center for Stroke Research Berlin, Charité—Universitätsmedizin Berlin, Corporate Member of Freie Universität Berlin, Humboldt-Universität zu Berlin, Berlin, Germany; 16 Department of Neurology with Experimental Neurology, Charité- Universitätsmedizin Berlin, 10117 Berlin, Germany

**Keywords:** Myasthenia gravis, Generalized myasthenia gravis, Prognostic biomarkers, Risk stratification, Acetylcholine receptor antibodies, Proteomics, Immune cell phenotyping, Disease activity, Complement activation

## Abstract

**Background:**

Myasthenia gravis has a variable disease course. Early identification of patients with a highly active disease is crucial to prevent myasthenic exacerbations and crisis, necessitating more aggressive immunotherapy. However, no biomarkers currently exist to identify patients at risk for highly active disease course.

**Objective:**

The PROGNO-MG study aims to identify blood-based biomarker signatures associated with a highly active disease course in immunotherapy-naïve patients with acetylcholine-receptor-antibody positive (AChR^+^) generalized Myasthenia gravis (gMG).

**Methods:**

This is an investigator-initiated prospective, multicentric, observational study. Seventy newly diagnosed immunotherapy-naïve AChR^+^ gMG patients will be enrolled and followed up for 24 months. Blood samples and clinical data will be collected biannually. Biomarkers (calprotectin, neurofilament light chain, ITIH3, complement activation, kappa free light chains, and exploratory proteome-analyses and immune cell phenotyping) will be measured and their association with clinical outcomes will be evaluated.

**Perspective:**

The results will provide evidence for prognostic biomarkers, supporting risk stratification and early individualized treatment for AChR^+^ gMG patients.

**Trial registration:**

The study is registered in the German clinical trial register (DRKS00035457).

## Introduction

Myasthenia gravis (MG) is a rare chronic autoimmune disorder marked by muscle weakness associated with fatigability. The clinical course varies widely, ranging from only ocular manifestations to generalized MG (gMG) with recurrent myasthenic crisis characterized by respiratory failure. Despite standard of care, disease burden remains high in many patients, especially with highly active disease course [1]. Consequently, novel therapeutic approaches have emerged, including C5 complement inhibitors and neonatal Fc receptor inhibitors, which have been approved as add-on therapies for patients with highly active acetylcholine-receptor antibody positive (AChR^+^) gMG and in the case of the FcRn inhibitor rozanolixizumab for muscle-specific kinase (MuSK) antibody-positive individuals [2].

The underlying pathophysiology involves autoantibodies targeting antigens at the motor endplate of the neuromuscular junction. IgG-antibodies directed against the acetylcholine-receptor are detected in approximately 80% of all patients, while antibodies against MuSK occur in 3-5% and lipoprotein receptor-related protein 4 (LRP4) account for approximately only 1% of patients. Notably, 10-15% of all patients remain seronegative [1, 3]. Antibody testing is crucial for establishing the diagnosis of MG, as it also influences treatment decisions, such as the consideration of thymectomy and the choice of immunotherapy [3]. However, the role of antibody levels as a biomarker to further guide treatment remain inconclusive and are not useful as a prognostic marker to identify patients at risk for a highly active disease course requiring escalation of immunotherapy to prevent myasthenic crisis or exacerbation [4]. In recent years, several other prognostic biomarkers have been proposed. Serum calprotectin and serum neurofilament light chain levels were shown to be significantly increased in MG patients, however, mild correlation to disease activity in a large patient population was only seen for calprotectin [5, 6]. Recently, proteomics-based approaches were useful in identifying single potential biomarker, e.g., inter-alpha-trypsin inhibitor heavy chain H3 (ITIH3) in AChR^+^ patients that correlated well with clinical disease activity [7], as well as clustering for proteome signatures that predict treatment response in subsets of MG patients [8]. In addition, comparative proteomic analyses have been useful in identifying additional candidate biomarkers to distinguish AChR+ MG from other neuroinflammatory diseases, further supporting the utility of proteomics-based approaches for biomarker discovery in MG [9]. Moreover, immune cell phenotyping and complement activation profiles have shown promise in exploratory studies [10–12]. Importantly, most existing biomarker studies in MG have been cross-sectional or included treatment-exposed populations, limiting their prognostic applicability. In this study, we seek to evaluate the prognostic value of these proposed biomarkers in a subset of AChR^+^ gMG patients with a highly active disease course, potentially supporting clinical decision-making and personalized care.

## Methods

### Aim of the trial

PROGNO-MG hypothesizes that gMG patients who will progress to a highly active disease variant show a distinct immune and biomarker signature prior to progression compared to patients showing a moderate disease course. This might enable personalized treatment decision in the future.

### Study description and study design

**Fig 1.**
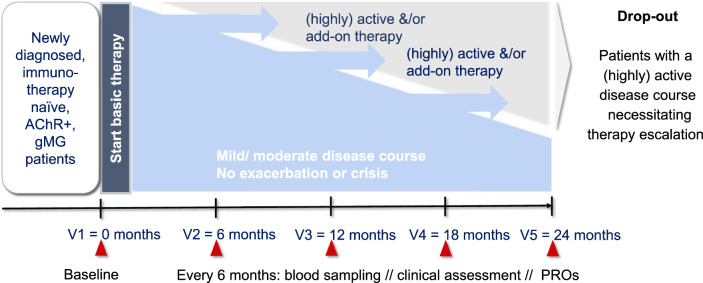
PROGNO-MG Study flow chart. Biannual study visits are planned for acetylcholine-receptor antibody positive (AChR^+^) generalized Myasthenia gravis (gMG) patients. Progression to a highly active disease course will lead to a drop-out and an unscheduled visit with clinical assessment, blood sampling and patient-reported outcome (PRO) assessment

PROGNO-MG is a prospective investigator-initiated, multicentric, non-interventional observational cohort study. The study is conducted across specialized MG centers in Germany. All enrolled patients will be followed for 24 months with standardized assessments every 6 months (Fig 1). PROGNO-MG is an observational study and participation will not impact on treatment choices or concomitant care.

### Eligibility criteria

Inclusion criteria are as following:

- Written informed consent
- Age 18-75 years
- Newly diagnosed AChR^+^ gMG (diagnosis <12 months) with one of the following: response to an acetylcholinesterase inhibitor or decrement after repetitive serial stimulation (outside M. orbicularis oculi) or increased jitter in single-fiber EMG
- Immunotherapy-naïve (corticosteroids and symptomatic therapy with pyridostigmine allowed)

Exclusion criteria:

- Prior immunosuppressive therapy (including azathioprine, mycophenolate mofetil, methotrexate, cyclosporine, tacrolimus) or targeted escalation therapies (including C5-inhibitor, FcRn-inhibitor, B-cell depletion like rituximab)
- Prior thymectomy or thymoma-associated MG
- Recent (≤3 months) use of intravenous immunoglobulins, plasma exchange, or immunoadsorption

### Arms and interventions

This prospective observational study will enroll 70 patients with gMG. Based on disease activity trajectories, we anticipate 10-15% of the participants will develop highly active disease within an observation period of two years. We will compare these individuals to the remaining 85-90% exhibiting a moderate disease course to identify associated biomarker signatures. Immunotherapy-naïve was defined as absence of prior treatment with long-term immunosuppressive or targeted immunotherapies. Corticosteroid treatment was permitted, as it represents standard first-line therapy in newly diagnosed generalized MG, and withholding treatment for study inclusion would not be ethically appropriate.

According to the German guideline for diagnosis and treatment of myasthenic syndromes, a highly active gMG, including so-called therapy-refractory MG were defined as [3]:

1. A moderate to high MGFA classification (≥ MGFA IIb) and/or at least two recurrent severe exacerbations or myasthenic crises requiring therapeutic intervention (e.g. IVIG, plasma exchange, or immunoadsorption) within one year of diagnosis, despite adequate disease-modifying and symptomatic treatment; **or**
2. Persistent, functionally relevant symptoms (≥ MGFA IIa) along with at least one severe exacerbation or myasthenic crisis within the past year, despite adequate disease-modifying and symptomatic treatment; **or**
3. Persistent, functionally relevant mild to moderate symptoms (≥ MGFA IIa) for more than two years, despite adequate disease-modifying and symptomatic treatment.

Statistical power was calculated to detect hazard ratios ≥3 for biomarkers predicting disease progression.

Patients will undergo a standardized assessment at baseline and every 6 months for overall 24 months (Table 1):

- Clinical assessments (e.g. MGFA [13], QMG [14], MGC scores [15]), including current immunotherapy and symptomatic therapy with reported side effects, thymectomy, comedications and comorbidities
- Patient-reported outcomes (MG-ADL [16], MG-QoL15r [17], Neuro-QoL Fatigue [18], Beck Depression Inventory [19], PASS [20], SSQ [21])
- Sampling for biomarker analysis
- Biomarker testing: calprotectin, neurofilament light chain, ITIH3, complement activation profiles, kappa free light chains
- Exploratory analyses: proteomics and immune cell phenotyping

**Table 1:** Summary of standardized assessment

Assessment	V1 (baseline)	V2 (6 months)	V3 (12 months)	V4 (18 months)	V5 (24 months)	Drop-out before regular study end
Clinical parameters (height, weight, ethnicity, family history, substance history, vaccination status)	X					
Clinical scores (QMG, MGFA, MGC)	X	X	X	X	X	X
Thymectomy status	X	X	X	X	X	X
Survey on adverse events		X	X	X	X	X
MG-specific therapy	X	X	X	X	X	X + planned intensified therapy
Comorbidities	X	X	X	X	X	X
Concomitant therapies	X	X	X	X	X	X
Patient reported outcomes (MG-ADL, MG-QoL15r; Neuro-Qol fatigue score, BDI, PASS, SSQ)	X	X	X	X	X	X
Biosampling (blood sampling)	X	X	X	X	X	X

MG = myasthenia gravis; QMG = quantitative myasthenia gravis score; MGFA = myasthenia gravis foundation of America clinical classification; MGC = myasthenia gravis composite score; MG-ADL = myasthenia gravis activities of daily living; myasthenia gravis quality of life revised; Neuro-Qol fatigue score = quality of life in neurological disorders; BDI = Beck´s depression inventory; PASS = patient acceptable symptom state; SSQ = single simple question

At baseline additional demographic data will be collected, including sex, age, body mass index, vaccination status and potential substance abuse. Unscheduled visits will be conducted in the event of disease exacerbations.

### Outcome measures

Primary endpoint is the development of a highly active disease course requiring escalation to add-on immunotherapy. Potential prognostic biomarkers (calprotectin, neurofilament light chain, ITIH3, kappa free light chains) as well as temporal differences for immune cell phenotyping, proteome analyses and complement activation profiles (circulating complement activation products and regulatory proteins, including C3b, iC3b, C4b, sC5b-9, Factor H, and Factor I) will be characterized. Secondary endpoints are demographic and clinical differences between highly active and moderate disease course and to assess the risk for myasthenic exacerbation/crisis of gMG patients within two years.

### Data and biosample management

Patient data will be pseudonymized locally on each study site and then be handled in electronic case report forms and submitted electronically via the open-source platform REDCap. The data monitoring committee is represented by F.S. and A.M., and will work independent of the sponsor. The sponsor of the study has no access to individual patient data. Pseudonymized biosamples will be forwarded for further processing to a biobank, enabling standardized processing and storage until further analysis. Standard operation protocols for biosample handling and shipping are implemented and followed by each study site. Missing data is handled based on the performed analysis, e.g., imputation for proteome analysis.

### Statistical analysis

The study is exploratory, and the number of cases is not sufficient to answer confirmatory questions. The study only serves to discover indications of possible correlations between biomarker levels and outcomes. Overall patient sample size is estimated based on a 10-15% of gMG patients progressing to a highly active disease course within two years for this explorative study [2, 3]. The sample size was calculated using a Cox regression for a *log Hazard Ratio on a covariate* with a standard deviation of 1. A sample size of n=70 provides a power of 81% for a regression coefficient of ≥ 1.099 (corresponding to a hazard ratio of ≥3) at a two-sided significance level of 5%. The calculation assumes an event rate of 12% (n = 8-9). The estimation accounts for potential confounding covariates in a multiple regression analysis, assuming an R^2^ of 0.2. The software nQuery Advisor 9 was used for sample size estimation. Cox proportional hazards models will be used to assess the relationship between biomarkers and disease progression. Time-dependent covariates and potential confounders will be adjusted in Cox regression models. Proteomic data will be analyzed using false discovery rate adjustments for multiple testing. Statistical analysis will be performed by the Institute of Biometry and Clinical Epidemiology Charité - Universitätsmedizin Berlin.

### Contacts

The responsible principal investigator is Frauke Stascheit, Department of Neurology, Charité- Universitätsmedizin Berlin.

### Perspective

PROGNO-MG will provide important, prospective clinical data on AChR^+^ gMG with highly active disease course and allow the evaluation of proposed biomarkers (calprotectin, neurofilament light chain, ITIH3, complement activation, kappa free light chains) in a well-defined immunotherapy-naïve gMG subset. It will further evaluate temporal immune cell phenotypes and proteomic profiles of these patients. Early identification of patients at risk for a highly active disease course may enable targeted therapeutic approaches to improve patient outcomes and reduce disease burden.

## Data Availability

Anonymized study data will be available upon reasonable request following publication of the study results.

## Abbreviations

AChR^+^: Acetylcholine-receptor-antibody positive
gMG: Generalized Myasthenia gravis
ITIH3: Inter-alpha-trypsin inhibitor heavy chain H3
LRP4: Lipoprotein receptor-related protein 4
MG: Myasthenia gravis
MG-ADL: Myasthenia gravis activities of daily LIVING
MGC: Myasthenia gravis composite
MGFA: Myasthenia gravis foundation of america
MG-QoL15r: Myasthenia gravis quality of life 15 revised
MuSK: Muscle-specific kinase
PASS: Patient acceptable symptom state
PRO: Patient-reported outcome
QMG: Quantitative myasthenia gravis
SSQ: Single simple question

## Declarations

### Protocol amendments

Version 1.0 of the study protocol. Any changes to the study protocol will be communicated immediately to all investigators and participants. Next to an update of the study registry, the investigators will publish an updated version of this manuscript.
